# Laparoendoscopic single-site surgery for deep infiltrating endometriosis based on retroperitoneal pelvic spaces anatomy: a retrospective study

**DOI:** 10.1038/s41598-023-38034-8

**Published:** 2023-07-04

**Authors:** Shoufeng Zhang, Hongxia Yu, ZhiYong Dong, Yao Chen, Wulin Shan, Wendi Zhang, Huiming Tang, Mengyue Chen, Weiwei Wei, Ruxia Shi, Bairong Xia, Jiming Chen

**Affiliations:** 1 Dalian Medical University, Dalian, 116000 People’s Republic of China; 2 Department of Obstetrics and Gynaecology, The Second Affiliated Hospital of Chongqing Medical University, Chongqing, 400010 People’s Republic of China; 3 Bengbu Medical College, Bengbu, 233030 People’s Republic of China; 4 Department of Obstetrics and Gynecology, The Affiliated Changzhou No. 2 People’s Hospital of Nanjing Medical University, Changzhou, 213000 Jiangsu Province China; 5 Department of Gynecology, The First Affiliated Hospital of USTC, Division of Life Sciences and Medicine, University of Science and Technology of China, Hefei, 230031 People’s Republic of China

**Keywords:** Anatomy, Diseases

## Abstract

Transumbilical single-port laparoscopy is widely used in gynecological surgery. However, it is rarely used in the treatment of deep infiltrating endometriosis due to its own shortcomings and the complex condition of deep infiltrating endometriosis. The study aims to introduce a transumbilical single-port laparoscopic surgery based on retroperitoneal pelvic spaces anatomy, which can complete the operation of deep infiltrating endometriosis more easily. A retrospective analysis of 63 patients with deep infiltrating endometriosis treated by transumbilical single-port laparoscopy using this method was conducted. The operation duration was 120.00 (85.00 ± 170.00) (35–405) min, the estimated blood loss was 68.41 ± 39.35 ml, the postoperative hospital stay was 5.00 (4.00–6.00) days, and the incidence of postoperative complications was 4.76% (3/63). 1 patient was found to have intestinal injury during operation, 1 patient had ureteral injury after operation, and 1 patient had postoperative pelvic infection, with a recurrence rate of 9.52%. The postoperative scar score was 3.00 (3.00–4.00) and the postoperative satisfaction score was 9.00 (8.00–10.00). In summary, this study demonstrates the feasibility of transumbilical single-port laparoscopic surgery for deep infiltrating endometriosis based on retroperitoneal pelvic spaces anatomy. Hysterectomy, adenomyosis resection, etc. are also feasible with this method, boasting more obvious advantages. This method can make transumbilical single-port laparoscopy more widely used in deep infiltrating endometriosis.

## Introduction

Deep infiltrating endometriosis (DIE) is a special type of endometriosis, accounting for about 10% of endometriosis, which occurs in women of a childbearing age. It refers to the growth of endometrial tissue with growth activity outside the uterine body and is often manifested as the infiltration of endometrial tissue into the subperitoneal depth of ≥ 5 mm or the muscularis propria of hollow organs, often combined with ovarian endometriosis cyst and peritoneal superficial endometriosis. These patients suffer noncyclic chronic pelvic pain, dysmenorrhea, deep dyspareunia, infertility, gastrointestinal symptoms, and lower urinary tract symptoms, seriously affecting their quality of life^1–3^.

Endometrial tissue is planted in the posterior pelvic cavity and can be planted in the ovary, uterosacral ligament (USL), rectocele, rectovaginal septum, sigmoid colon, rectum, bladder, and ureter, very few endometrial tissues can involve extra-abdominal organs such as the lungs^4–6^. DIE experiences infiltrative growth in the posterior pelvic cavity, resulting in dense fibrosis caused by severe pelvic adhesions and normal anatomical distortion. Hormonal drug therapy is the first choice for symptomatic women; Surgical treatment may be recommended for women who are unresponsive, intolerant, or have control indications to medication, or for women with severe intestinal or urinary tract stenosis related to the disease. Therefore, the treatment of DIE is mainly surgical treatment combined with long-term postoperative drug management^3^. The purpose of surgical treatment is to remove the lesion, restore anatomy, and promote fertility. Laparoscopic surgery is the main surgical method for the treatment of DIE. Compared with open surgery, laparoscopic surgery has obvious advantages, such as less surgical trauma, less postoperative pain, shorter hospitalization time, and other minimally invasive advantages. Meanwhile, the magnified field of view can better identify endometriosis lesions, which is conducive to complete resection, however, surgical treatment may increase the risk of ureteral, bladder, and rectal injuries. At present, the main surgical method is multi-port laparoscopic surgery. Conventional multi-port laparoscopic surgery produces 3–4 surgical scars on the abdominal wall, and the aesthetics is affected to a certain extent^1–3,7,8^.

With the advancement of minimally invasive surgical techniques, laparoendoscopic single-site surgery (LESS) develops fast in gynecological surgery, with advantages including a lower number of abdominal wall surgery scars and better-looking surgery incisions, and its therapeutic effect has been proved in a variety of gynecological benign and malignant diseases^9,10^. However, due to the serious adhesion of the posterior pelvic cavity, the surgical operation space of DIE is narrow, enlarging the defects such as the lack of LESS operation triangle, mutual interference between instruments, and a narrow visual field^9^. Meanwhile, DIE is often associated with multiple system diseases such as urinary system and digestive system. It remains a huge challenge to remove the lesions safely and thoroughly. These factors limit the application of LESS in deep infiltrating endometriosis.

With the development and improvement of modern anatomy, retroperitoneal pelvic spaces anatomy is widely used in colorectal surgery, urology, and gynecological surgery^11–13^. This non-vascular area helps achieve efficient and accurate resection of lesions and reduces the damage of normal tissues. DIE surgery needs to separate the adhesive tissues and organs around the lesion, and then remove the infiltrating lesions, which is closely related to retroperitoneal pelvic spaces anatomy. We hope that this concept of surgical anatomy of transumbilical single-port laparoscopic surgery for DIE surgery can optimize the surgical path, reduce the difficulty of operation, shorten the operation duration, reduce intraoperative blood loss, and reduce the incidence of surgical complications, to ensure complete resection of lesion, relieve pain, and reduce the recurrence.

The purpose of this study is to propose an improved surgical method for the treatment of DIE using LESS based on retroperitoneal pelvic spaces anatomy to reduce the difficulty of surgery and facilitate understanding and learning.

## Method

This study is a single-center retrospective study that analyzed patients who underwent LESS for DIE using retroperitoneal pelvic spaces anatomy from January 2017 to January 2021. All patients were performed by a minimally invasive gynecologist who was proficient in LESS and were diagnosed with DIE during intraoperative or postoperative pathological examination. The Affiliated Changzhou No. 2 People's Hospital of Nanjing Medical University approved the study (No. [2021] YLJSD017). Before conducting this study, our team had successfully operated on 7 patients with pelvic DIE using this method. The patients included in the study were free to choose between conventional multi-port laparoscopy and LESS for surgical treatment. All patients signed informed consent before surgery. We collected all the clinical data of the included patients through the electronic case system and obtained the written informed consent of the patients before collecting their clinical data.

### Retroperitoneal pelvic spaces

The retroperitoneal pelvic spaces refers to the potential avascular space filled with loose connective tissue fascia or fat formed between the fascia covering the muscles of the pelvic wall and the visceral fascia covering the pelvic organs. In this study, the surgical procedure was based on the pararectal space (PRS) and the rectovaginal space (RSV). The two spaces are of great significance for the separation of important tissues and organs, and the safe and thorough resection of lesions in the operation of DIE.

#### Pararectal space

Also known as pelvic rectal space, the pararectal space is located between the two sides of the rectum and the lateral wall of the pelvis. The superior border is the peritoneum of the lateral rectal fossa, the inferior border is the levator ani muscle, the medial border is the rectal fascia sheath, the lateral border is the internal iliac vessels and the pelvic wall, the ventral side is the main ligament, and the dorsal side is the presacral fascia or rectal ligament. The longitudinal ureter divides the PRS into the medial pararectal space (MPS) (Okabayashi's space) and the lateral pararectal space (LPS) (Latzko's space). The outer side of the lateral pararectal space is the pelvic side wall, the inner side is the ureter, and the uterine blood vessels pass through this space. This space is generally used for pelvic lymph node resection in gynecological malignant tumor surgery and deep infiltrating endometriosis of the pelvic side wall resection. The lateral side of the MPS is the ureter, and the medial side is the rectum or the USL. The lower abdominal plexus and pelvic splanchnic nerve are in the deep part of the MPS. This space is particularly important for the resection of endometriosis lesions of free ureter, rectum, and USL in DIE surgery and for DIE surgery with nerve preservation surgery^11,13^.

#### Rectovaginal space

Avascular space was formed between vagina and rectum. The anterior boundary was the posterior wall of vagina, the posterior boundary was the anterior wall of rectum, the head end was the peritoneal reflection of the uterine rectal fossa and the head end of the bilateral USL, and the tail end was the levator ani muscle. The fascia covered by the outer layer of the posterior vaginal wall and the visceral fascia of the rectum fuse with each other from bottom to top along the RSV to form a frontal fascia plate filled with loose connective tissue, which is called the rectovaginal septum, also known as the Denonvilliers fascia. Pelvic DIE surgery can be through both sides of the USL and the opened uterus into the concave rectal vascular space, which is conducive to the safe separation of the vagina and rectum and plays an important role for the removal of rectovaginal septum endometriosis^11,13^.

### Surgical techniques

The operation is carried out in 10 steps. 1. Separate pelvic wall adhesions; 2. Remove ovarian cysts and suspend ovaries; 3. Free ureter; 4. Open PRS; 5. Dissociate USL and remove DIE nodules from USL; 6. Remove adenomyosis of the posterior uterine wall; 7. Open RSV; 8. Remove intestinal endometriosis nodules; 9. Remove rectovaginal septum endometriosis nodules; 10. Examine bladder and rectal injuries. The above surgical steps are not mandatory, and a personalized surgical plan should be formulated according to the pelvic adhesion of each patient.

The surgical channel of LESS was established through a 1.5–2.5 cm umbilical incision. Enter the abdominal cavity, expose the abdominal cavity and pelvic cavity with 30° laparoscope to evaluate the feasibility and difficulty of the operation. In the event of severe pelvic adhesions, which makes LESS difficult, the surgery can be converted to conventional multi-port laparoscopy or open surgery. An additional 5 mm operating hole can be added during the operation according to the difficulty. Dissect and remove the lesion using bipolar coagulation, ultrasonic scalpel, or sharp scissors. A multidisciplinary surgical team led by gynecologists and supported by colorectal surgeons and urologists was established.

The sigmoid colon and mesangial tissue adhered to the pelvic lateral wall were separated, and the superficial endometriosis lesions on the peritoneum of the pelvic lateral wall were electrocoagulated or removed. The ovarian endometriosis cyst adhered to the surrounding tissue was freed from the pelvic side wall, broad ligament, ovarian fossa, or uterine wall. Ovarian endometriosis cysts were incised, and all old blood in ovarian endometriosis cysts was aspirated and rinsed using a negative pressure aspirator. The cyst wall was stripped along the incision of the cyst, and the cyst wall with mild infiltration and obvious boundary with normal ovarian tissue was easily removed. The cystic wall with blurred boundaries caused by deep infiltration of the lesion and close adhesion with the ovary should retain normal ovarian tissue as much as possible during stripping to reduce the impact on the endocrine and reproductive function of the ovary after surgery. The remaining ovarian tissue was sutured with 3–0 absorbable sutures for hemostasis and ovarian molding surgery, and the molded ovary was suspended on the anterior abdominal wall or pelvic lateral wall using a straight or curved needle.

**Figure 1:**
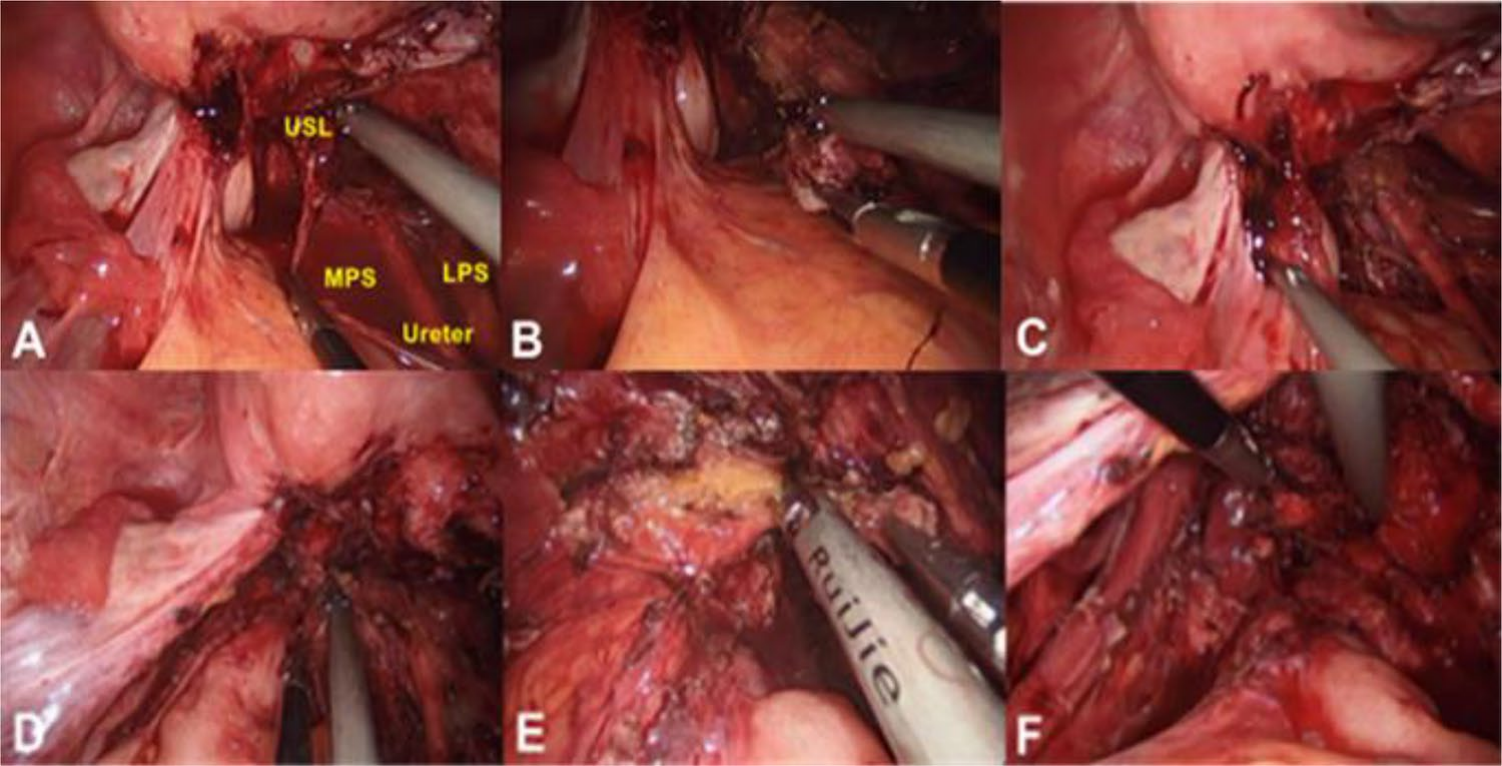
(**A**) Free the ureter, open the lateral pararectal space and medial pararectal space on both sides of the ureter. (**B**) Remove the left uterosacral ligament endometriosis nodule; (**C**) Open the rectum and uterine cavity, and chocolate-colored liquid flow out; (**D**) Divide the endometriosis nodule into two parts II; (**E**) Remove endometriosis nodules on the rectal side. The yellow tissue under the nodules was adipose tissue (safety boundary); (**F**) Remove the endometriosis nodules on the posterior vaginal wall. (MPS, Medial pararectal space; LPS, Lateral pararectal space).

Identify the ureter from the normal peritoneum where the ureter crosses the iliac vessels and open the abdomen to free the ureter. Free along the direction of the ureter to the lateral rectal fossa. Open LSP on the lateral side of the ureter, check the presence of pelvic wall endometriosis lesions, uterine artery separation, and then push the ureter to LSP. Open the MSP and free the ureter to the USL along the MSP (Fig. 1A). When dissociating and releasing the ureter, pay attention to the ureteral variation and maintain the integrity of the ureteral blood vessels and mesangium. Make further anatomical separation to the deep pelvic here, and the lower abdominal plexus and pelvic visceral nerve will show. Carefully identify whether the nerve fibers are infiltrated by endometriosis lesions. Extrapolate the nerve tissue and ureter and pull the USL medially to remove the USL endometriosis nodules (Fig. 1B). Dissociate the contralateral ureter and remove the contralateral USL endometriosis nodule in the same way.

DIE is closely related to adenomyosis. Most patients have adenomyosis of the posterior wall of the uterus. The posterior wall of the uterus often forms dense adhesions with the anterior wall of the rectum. Through the MSP on both sides of the rectum, the adhesion can be separated by removing part of the posterior wall of the uterus on the serosal side of the posterior wall of the uterus, and the lesion on the rectal side can be removed close to the serosal side of the rectum. Subsequently, adenomyosis of the posterior wall of the uterus was removed, and the uterus was sutured and reshaped using absorbable barbed sutures.

After the excision of all visible pelvic endometriosis lesions, continue to dissociate both sides of the MSP to the left and right sides of the USL broken end and rectal anterior wall. Determine the uterine rectal depression and RSV position, open the closed uterine rectal depression (Fig. 1C), enter the RSV, and perform blunt separation of RSV loose connective tissue until the nodule lesions are reached. For rectovaginal diaphragmatic endometriosis nodules ≥ 1 cm in diameter, free the lesions from both sides to the levator ani muscle, and then cut the nodules in half from the middle to increase the space required for subsequent resection (Fig. 1D). Rectal endometriosis lesions were removed first, and the lesions infiltrating the intestinal serosal layer or superficial muscular layer were removed. The lesions were pulled to the vaginal side to increase the safety gap between the lesions and the anterior wall of the rectum. The adipose tissue on the surface of the rectum was removed as a safe boundary near the anterior wall of the rectum (Fig. 1E). Absorbable sutures were used to repair and strengthen the weak intestinal wall. For lesions infiltrating the entire intestinal wall, a colorectal surgeon performed intestinal butterfly resection or segmental bowel resection and anastomosis. Subsequently, the posterior vaginal wall lesion nodules were pulled to the rectal side, close to the posterior vaginal wall resection. For deep infiltration lesions, part of the vaginal wall can be removed to achieve complete resection of the lesion and reduce the postoperative recurrence rate. Absorbable sutures should be used to close the vaginal wall gap. Rectovaginal septum endometriosis lesions less than 1 cm were directly removed. The assistant manipulated the uterine lifter to lift the posterior wall of the vagina upwards. The surgeon clamped the anterior wall of the rectum and pulled it downwards to release the lesion from the anterior wall of the rectum (Fig. 1F). The suspended ovary was placed in the ovarian fossa and the pelvic cavity was rinsed.

The suspended ovary was placed in the ovarian fossa and the pelvic cavity was rinsed. Whether the bladder and rectum were damaged during the operation was determined by injecting methylene blue reagent into the bladder and performing rectal inflation test. Adhesion material was placed on the surgical wound to prevent postoperative adhesion, and a one-time drainage tube was placed through the umbilical incision to end the operation. Patients were given antibiotics to prevent infection and heparin to prevent thrombosis after operation.

### Postoperative treatment and follow‑up

**Figure 2:**
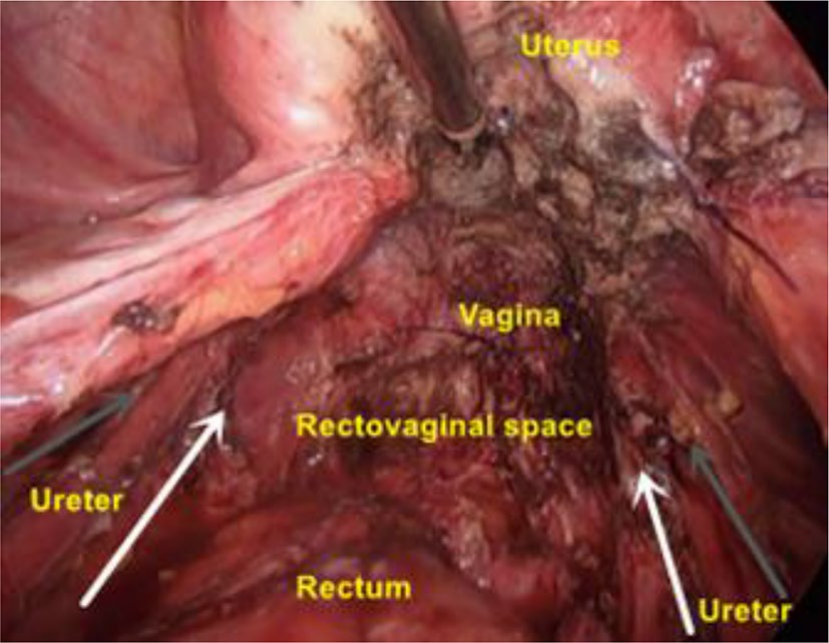
Resection of all pelvic endometriosis lesions in a patient with stage IV (rAFS score: 94) severe DIE resulted in restoration of normal pelvic structures due to adhesion and disruption, as shown in the figure. The white arrow indicates the medial pararectal space, and the gray arrow indicates the lateral pararectal space.

According to the needs of postoperative pregnancy, progesterone, gonadotropin-releasing hormone agonists, contraceptives, and other drugs were used for long-term management and treatment to reduce the recurrence rate (Fig. 2). Patients were examined once a month after surgery for 6 months, then once every 6 months for 3 years, and finally once a year.

### Outcomes

The clinical data obtained from electronic cases mainly include demographic data, perioperative data, and follow-up data. It includes age, body mass index (BMI), CA125, previous history of endometriosis surgery, symptoms, combined adenomyosis, operation duration, estimated blood loss, Clavien-Dindo (C-D) classification of surgical complications, hospitalization time (from the first day after surgery to discharge), converted surgical methods, postoperative scar score, patient satisfaction with surgery, and recurrence. Endometriosis was classified by revised American Fertility Society Scoring (r-AFS) and the revised ENZIAN score (r-ENZIAN) during surgery^14^. Recurrence includes symptoms suggested recurrence, vaginal ultrasound suggested recurrence, and symptoms and vaginal ultrasound results suggested recurrence. Six months after the operation, a non-surgeon physician scored the patient's umbilical incision scar following the vancouver scar scale (VSS). At the same time, the visual analog scale represents patients’ overall satisfaction with surgery (VAS for surgery) was used to evaluate the satisfaction of the patient 's surgical results. The subjective satisfaction ranged from 1 to 10 points (0 points represented 'very dissatisfied' and 10 points represented 'very satisfied').

## Statistical analysis

All results were analyzed using SPSS 26.0 (IBM Corp, Armonk, NY, USA). The Shapiro–Wilk test was used to examine the normality of descriptive data distribution. Measurement data conforming to normal distribution are expressed as the mean (standard deviation); and measurement data not conforming to normal distribution are expressed as median (interquartile range [IQR]). Categorical variables are expressed in cases and percentages.

## Results

From January 2017 to January 2021, a total of 63 patients with pelvic DIE successfully underwent LESS. Among them, the intraoperative operation of 6 patients was difficult due to severe pelvic adhesions and the operation was successfully completed after an additional 5 mm auxiliary operation hole was added.

**Figure 3:**
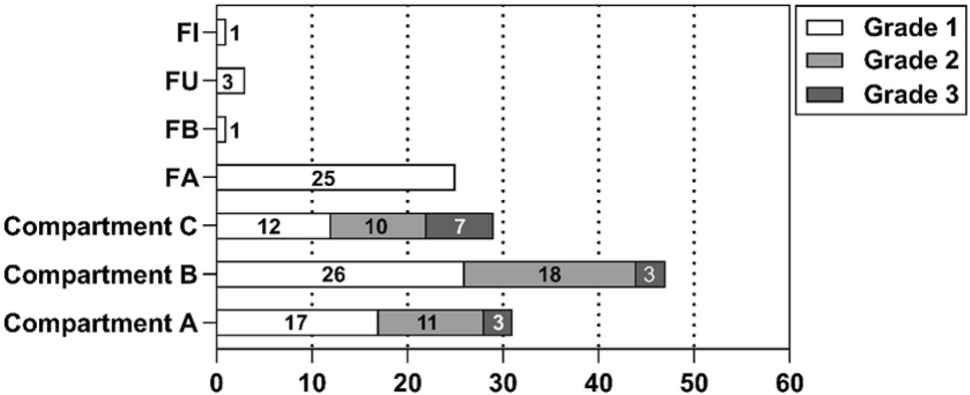
This figure shows the r-Enzian classification of 137 lesions found during surgery in 63 patients with DIE. Compartment A, Rectovaginal septum and vagina; Compartment B, Sacrouterine ligament to pelvic wall; Compartment C, Rectum and sigmoid colon. FA, Adenomyosis; FB, Involvement of the bladder; FU, Intrinsic involvement of the ureter; FI, Bowel disease cranial to the rectosigmoid junction; Grade 1, Invasion < 1 cm; Grade 2, Invasion 1–3 cm; Grade 3, Invasion > 3 cm.

The baseline characteristics of the 63 patients in this study are shown in Table 1. Among them, 14 patients needed reoperation because of recurrence after previous endometriosis surgery. The urinary tract symptoms of the patients were mainly hematuria in 2 cases, frequent urination in 2 cases, and mild ureteral dilatation in 2 cases. The gastrointestinal symptoms of the patients were mainly constipation in 5 patients, diarrhea in 4 patients, and defecation pain in 1 patient. Meanwhile, 25 cases of DIEpatients had varying degrees of adenomyosis. Stage I 4.00 (3.25–4.00), Stage II 10.86 ± 2.34, Stage III 29.00 ± 6.92, and Stage IV 65.53 ± 12.36. All lesions of the 63 patients were classified according to r-Enzian as shown in Fig. 3.

**Table 1:** Patient characteristics of 63 patients.

Characteristic	Value
Age, years, mean ± SD	31.25 ± 5.81
BMI, kg/m^2^, mean ± SD	22.62 ± 2.79
CA125, (U/mL), mean ± SD	114.74 ± 65.29
Previous history of endometriosis surgery, number (n, %)	14(22.22%)
Symptom	
Pain (n, %)	55(87.30%)
Urinary symptoms (n, %)	6(9.52)
Gastrointestinal symptoms (n, %)	10(15.87)
Infertility (n, %)	9(14.28)
Adenomyosis (n, %)	25(39.68)
rAFS (n, %)	
Stage I	4(6.35%)
Stage II	7(11.11%)
Stage III	20(31.75%)
Stage IV	32(50.79%)

BMI, Body mass index; CA 125, Carbohydrate antigen 125. Pain symptoms include noncyclic chronic pelvic pain, dysmenorrhea, and deep dyspareunia. Gastrointestinal symptoms include with menstrual diarrhea, constipation, defecation pain or blood in the stool. Urinary symptoms include urinary pain, urinary frequency, urgency, hematuria, ureteral dilatation or hydronephrosis.

During the study period, 45 patients underwent cystectomy for ovarian endometriotic cysts, 22 patients underwent adenomyosis-sparing hysterectomy, and 3 patients without fertility requirements underwent hysterectomy for adenomyosis. The operation duration of the patients was 120.00 (85.00–170.00) min, the estimated blood loss was 68.41 ± 39.35 ml, the postoperative hospital stay was 5.00 (4.00–6.00) days, and the incidence of postoperative complications was 4.76% (3/63). According to the Clavien-Dindo classification of surgical complications, 1 patient was Clavien-Dindo Grade II, with postoperative pelvic infection, and was discharged after antibiotics was used. 2 patients were Clavien-Dindo Grade III. 1 patient was found to have intestinal injury during the operation and was cured after intestinal repair during the operation. 1 patient was found to have ureteral injury after the operation, which was not found during the operation, and recovered after double J stent drainage. The surgical results are shown in Table 2.

**Table 2:** Operative outcomes of 63 patients.

Characteristic	Value
Operation time, minutes, mean ± SD	120.00(85.00–170.00)
Estimated blood loss, milliliters, mean ± SD	68.41 ± 39.35
Complications according to Clavien-Dindo classification (n, %)	3(4.76%)
Clavien-Dindo grade I–II	1
Clavien-Dindo grade III–V	2
Length of hospital stay, nights, mean (SD)	5.00(4.00–6.00)
Ovarian endometriosis cyst removal (n, %)	45(71.43%)
Uterine—preserving adenomyectomy (n, %)	21(33.33%)
Hysterectomy (n, %)	4(6.35%)
Others (n, %)	5(7.94%)

Others: 3 patients had uterine fibroids, 1 patient had rudimentary horn uterus, and 1 patient had uterine septum.

The postoperative follow-up time was 22.90 ± 5.45 months. During the follow-up period, a total of 6 (9.52%) patients were found to have postoperative recurrence. Among them, 3 patients had recurrence of pain symptoms, 2 patients had recurrence of ovarian endometriosis cyst according to vaginal ultrasound results, and 1 patient had recurrence of pain symptoms combined with vaginal ultrasound. The postoperative VSS was 3.00 (3.00–4.00), and the patient satisfaction score was 9.00 (8.00–10.00). No incisional hernia occurred in all patients during postoperative follow-ups (Table 3).

**Table 3:** Follow-up examination data of 63 patients after surgery.

Variables	Value
The length of follow-up, months, mean ± SD	22.90 ± 5.46
VSS	3.00(3.00–4.00)
VAS of surgery	9.00(8.00–10.00)
spontaneous pregnancy (n, %)	5(55.56)
Recurrence, number (n, %)	6(9.52)
recurrence of symptoms (n, %)	3(50)
Transvaginal ultrasonography suggested recurrence (n, %)	2(33.33)
recurrence of symptoms and transvaginal ultrasonography suggested recurrence (n, %)	1(16.67)

VSS, Vancouver scar scale; VAS, VAS for surgery = visual analog scale represents patients’ overall satisfaction with surgery.

## Discussion

Transumbilical single-port laparoscopy has been rapidly and widely applied in the gynecological surgery because of its unique minimally invasive advantages without obvious scars on the abdominal wall^15^. DIE patients often have severe pelvic adhesions and multi-system involvement, coupled with problems such as complicated surgical operation, long operation time, intraoperative bleeding, and high rate of conversion to other surgical methods, LESS not a wise option^16^. We designed a suitable surgical procedure based on the characteristics of LESS and DIE.

The concept of retroperitoneal pelvic spaces anatomy originated from Werthem and Okabayasi's radical hysterectomy for cervical cancer and nerve-sparing radical hysterectomy for cervical cancer to shorten the operation duration, reduce the amount of bleeding, reduce the incidence of surgical complications, and improve the survival rate of cancer patients^17^. With the improvement of gynecological pelvic fascia anatomy system, this anatomical concept has been gradually applied to other complex gynecological operations^13^. Different from radical surgery for cervical cancer, although DIE is a benign disease and does not require pelvic lymph node dissection, DIE causes dense adhesions and fibrosis around the lesion, resulting in the disappearance of the original anatomical markers and levels. The premise of safe resection of the lesion is to free and expose the normal non-vascular space around the lesion with important organs, blood vessels, and nerves, and to remove the lesion to the greatest extent.

Retroperitoneal pelvic spaces associated with DIE surgery includes pararectal space, rectovaginal space, retrorectal space (presacral space), retropubic space, bladder uterine space, bladder vaginal space, paravesical medial space, and the fourth space^13^. Meanwhile, retroperitoneal pelvic spaces anatomy is conducive to the preservation of nerve function in DIE surgery. By entering the MPS, we can identify the lower abdominal plexus and pelvic visceral nerves. These nerves dominate the physiological functions of the bladder, cervix, upper vagina, and rectum. Preserving nerve function helps protect defecation, urination, and sexual functions and reduce the incidence of neurogenic urination dysfunction^18,19^, to preserve pelvic organs' function it is mandatory to preserve hypogastric nerve and pelvic plexus lining in the postero-lateral parametrium. This surgical method is also conducive to restoring the original adhesion and chaotic anatomy to a normal physiological structure, which is conducive to the recovery of postoperative reproductive function in infertile patients. After our surgical method improvement, the operation duration was 140.81 ± 74.66 min, and the estimated intraoperative blood loss was 68.41 ± 39.35 ml, similar to what Saget E et al. reported, that is, the operation duration of robot-assisted laparoscopic surgery was 138 ± 75 min, and the estimated intraoperative blood loss was 70 ± 107 ml. The incidence of surgical complications was 4.76% (3/63). The natural pregnancy rate after surgery was 55.56% (5/9), higher than the reported 43.64% (24/55), which may be related to the small sample size of infertility patients in this study^20^. The recurrence rate was 9.52% (6/63), The follow-up time was 22.90 ± 5.46 months, the low recurrence rate may be related to the short follow-up time^21^.

Preoperatively, the feasibility of LESS was comprehensively evaluated based on the patient's symptoms, signs, imaging studies, ureteroscopy or colonoscopy examination results. It is very important for surgeons to conduct vaginal examination and rectal examination before operation to judge the depth of lesion infiltration and formulate surgical methods. The feasibility of LESS involving complex urinary tract and intestinal tract should be jointly evaluated by gynecologists, urologists, and colorectal surgeons. Transumbilical single-port laparoscopy can be used in urology and colorectal surgery related to DIE, but it is rarely used currently. The traditional multi-port laparoscopy or robot-assisted laparoscopic surgery is still recommended for complex multidisciplinary surgery to reduce the conversion rate of surgery.

In DIE, the ureter is often obstructed and tortuous due to the traction of dense fibrous tissue or ureteral involvement, which makes the ureter lose its normal anatomical structure. We recommend that the ureter be identified and dissected at the normal position of the ureter across the iliac vessels, and the ureter is safely freed through the open PRS. Kondo W and Cabrera R et al. proposed different strategies for treating rectovaginal septum endometriosis^22,23^. In this study, according to the size of the rectal vaginal septum nodule, different surgical methods were selected. For the lesions with rectal vaginal septum endometriosis ≥ 1 cm, first free both sides of the nodule, then divide the nodule into two parts from the middle to release the operating space, first deal with the lesions on the rectal side, and finally deal with the lesions on the vaginal side; The lesions with endometriosis of the rectal vaginal septum < 1 cm were treated first on the rectal side and then on the vaginal side. Because the treatment of rectovaginal septum endometriosis is often the last part of the operation, the long-term surgery may lead to fatigue of the operator and decrease of operation ability. Treatment of the rectal side first helps reduce the rectal injury caused by fatigue. Compared with rectal injury, gynecologists are more confident in dealing with vaginal injury. When the lesion obviously infiltrates the vaginal wall or penetrates the vaginal wall, the vaginal wall may be damaged or partial vaginal resection may be performed to improve the resection rate of the lesion. However, poor wound healing or fibrosis after surgery may lead to difficulty in sexual intercourse^24^, which in turn affects fertility.

Appropriate surgical techniques are also the key to the success of LESS. After the removal of ovarian endometriosis cyst, the ovary is suspended on the anterior abdominal wall or peritoneum to provide the tension required for the operation space, vision, and anatomy of the subsequent free ureter. The “chopsticks technique” proposed by Wang Y et al. can reduce the difficulty of complex surgical operations by addressing the lack of surgical triangles and mutual interference of instruments in LESS^9^, facilitating fine anatomy of important organs and tissues in DIE surgery. When freeing blood vessels, ureters and intestines, meticulous operation also reduces the bleeding of mesangial injury, and blood is easy to accumulate in the open space, affecting the exposure of surgical field. Suture is a challenging issue for surgeons using LESS, especially when suturing the vaginal gap. The narrow surgical field and operation space affect the knotting. A coil can be made in the pelvic cavity, and then the coil is pushed into the position where suture knot is needed. Second, for patients undergoing adenomyosis resection or hysterectomy, the use of absorbable suture can significantly shorten the suture time and reduce the amount of bleeding. For patients with severe pelvic adhesions or difficult surgery, an additional 5 mm operating hole can be used for auxiliary operation. The operating hole is usually located at the middle and outer 1/3 junction of the umbilical cord and the left or right anterior superior iliac spine^25^. In our study, 6 patients achieved good results in this way.

Our research advantage is that the application of LESS in the surgical treatment of DIE broadens the scope of LESS application and proposes an improved surgical procedure based on retroperitoneal pelvic spaces anatomy, with good results achieved. We believe that this may be a very suitable standard method for LESS surgery for treating DIE. Meanwhile, we recognized some limitations in our study, including the small sample size, the single-center retrospective study, and a lack of control group. Since our surgeon is a doctor who is proficient in LESS operation, the surgeon's LESS operation experience is also an important contributing factor. In the future, multi-center, multi-sample, prospective studies will be conducted, and the learning curve of the surgical approach will be studied to try multidisciplinary surgery for complex DIE and let more patients benefit from the minimally invasive advantages of LESS.

## Conclusion

In summary, LESS for DIE based on retroperitoneal pelvic spaces anatomy may be safe and feasible. This improved surgical procedure is conducive to streamline the operation steps, reduce the difficulty of surgery, shorten the operation duration, and reduce blood loss and complications. The surgical procedure is not mandatory. It should respect patients' needs and be based on lesion size and location and the surgeon's operation skills.

## Data Availability

The datasets used and/or analysed during the current study are available from the corresponding author upon reasonable request.
